# The Association Between Leucine and Diabetic Nephropathy in Different Gender: A Cross-Sectional Study in Chinese Patients With Type 2 Diabetes

**DOI:** 10.3389/fendo.2020.619422

**Published:** 2021-02-09

**Authors:** Xiaoqian Gao, Ruiqin Hou, Xin Li, Xing-Hua Qiu, Hui-Huan Luo, Sheng-Lin Liu, Zhong-Ze Fang

**Affiliations:** ^1^ Department of Toxicology and Sanitary Chemistry, School of Public Health, Tianjin Medical University, Tianjin, China; ^2^ Department of Blood Transfusion, Peking University People’s Hospital, Beijing, China; ^3^ State Key Joint Laboratory of Environmental Simulation and Pollution Control, College of Environmental Sciences and Engineering, Peking University, Beijing, China; ^4^ Department of Laboratory Center of Tianjin Xiqing Hospital, Tianjin, China; ^5^ Tianjin Key Laboratory of Environment, Nutrition and Public Health, Tianjin, China

**Keywords:** leucine, diabetic nephropathy, metabolism, type 2 diabetes, gender

## Abstract

**Objective:**

This study aimed to evaluate how leucine are associated with diabetic nephropathy (DN) in type 2 diabetes (T2D) patients and the gender difference of this association.

**Methods:**

We retrieved 1,031 consecutive patients with T2D who meet the inclusion and exclusion criteria from the same tertiary care center and extracted clinical information from electronic medical record. Plasma leucine was measured by liquid chromatography-mass spectrometer. Restricted cubic spline (RCS) was conducted to examine potential non-linear relationship between leucine and the risk of DN. Logistic regression was used to obtain odds ratio (OR) and confidence interval (CI). Additive interaction was used to estimate the interaction effect between leucine and gender for DN.

**Results:**

We found there was a negative correlation between leucine and the risk of DN. After stratifying all patients by gender, this relationship only remained significant in women (OR:0.57, CI:0.41–0.79).

**Conclusions:**

In conclusion, T2D patients with high levels of leucine have a lower risk of developing DN in female.

## Introduction

Diabetic nephropathy (DN), one of the common complications of diabetes, greatly increases mortality and medical expenses in type 2 diabetes (T2D) patients (1, 2). DN increased enormous societal burden, as it also amplifies the risk of other diabetes complications including cardiovascular disease, heart failure, infections (3–7). Since DN is a kind of progressive disease, it is important to search for new approaches that can effectively forecast and prevent the onset of DN. However, noninvasive available markers for accurate prediction and diagnoses of DN in diabetic patients are lacking now (8). It is worth noting that many studies have found the burden of diabetes is different in different genders (9, 10). For example, women have higher mortality rate for diabetes-related deaths, including DN (9). One may speculate that there is maybe something different in pathology and predictors of DN between men and women.

The development of new technologies allows the high-throughput profiling of metabolic status from a blood specimen (metabolomics). More and more researches focused on exploring whether metabolite profiles affect the onset and development of DN *via* combining epidemiology and metabolomics (11–14).

Insufficient insulin secretion, one of the risk factor of DN, was found to be closely associated with plasma amino acids level (15). Different from other amino acids, leucine, one of branched-chain amino acids (BCAAs), is catabolized in skeletal muscle which is important organ for the regulation of blood glucoses (16, 17). In addition to the metabolic roles, leucine was reported in many studies as the regulator of the mechanistic target of rapamycin (mTOR) pathway which could affect insulin secretion (18). In animal tests, leucine was found attenuate DN progression (19, 20). Although physiological investigations suggested great insight into mechanisms, yet they do not conclusively illustrate that such mechanisms work at the population level in humans. In fact, there was only some population studies expounding the relationship between leucine and diabetes, but it is still a lack of population evidence about leucine and the risk of DN (21).

In this study, we established a cross-sectional study in a Chinese population, and aimed to 1) evaluate association between plasm leucine and risk of DN; 2) examine whether this association will change in different gender.

## Materials and Methods

### Study Method and Population

We retrieved electronic medical records with available metabolite data from the First Affiliated Hospital of Liaoning Medical University (FAHLMU) in Jinzhou, Liaoning Province, China. The inclusion criteria were: diagnosed as T2D or treated with antidiabetic drugs. Exclusion criteria were: 1) ages under 18 years old; 2) lacked height, weight and blood pressure. A total of 1898 T2D patients were preliminarily enrolled into this study. According to the exclusion criteria, 866 patients were excluded as under 18 years and 1 was excluded as lacked height, weight and blood pressure. Finally, a total of 1031 research subjects were included the present study. Among them, 188 DN patients were diagnosed. The diagnosis and classification of type 2 diabetes mellitus in the present study were based on the standard published by World Health Organization(WHO) or treated with antidiabetic drugs (22). The diagnostic criteria for diabetic nephropathy was based on the standards of care for type 2 diabetes (23).

The Ethics Committee for Clinical Research of FAHLMU approved the ethics of the study, and informed consent was waivered due to the retrospective nature of the study, which is consistent with the Declaration of Helsinki.

### Data Collection and Clinical Definitions

The data retrieved from the electronic medical records for both groups contained demographic and anthropometric information, as well as current clinical factors, medications and complications of diabetes. Demographic included gender, current status of smoking and alcohol consumption. Anthropometric measurements yielded information included height, weight, systolic blood pressure (SBP) and diastolic blood pressure (DBP). Clinical parameters contained glycosylated hemoglobin((HbA1c), triglyceride (TG), high-density lipoprotein cholesterol (HDL-C), low-density lipoprotein cholesterol (LDL-C), urinary creatinine (UA), serum creatinine (SCR). Details of medication use were documented, including oral anti-diabetic drugs, insulin, and lipid lowering drugs, statins. Duration of diabetes and diabetic nephropathy were recorded.

In hospitals, anthropometric indices were measured by using standardized procedures. Participants were allowed to wear light clothes and no shoes. Height and weight were measured to the nearest 0.5 cm and 0.1 kg respectively. Blood pressure was measured behind the right arm of an adult cuff using a standard mercury sphygmomanometer and post-measurement at an appropriate size, after a 10 min rest in a sitting position. Age was calculated from the date of birth to the date of hospitalization or medical examination, and was calculated in years. The body mass index (BMI) was calculated as the ratio of weight(kg) to squared height(meters) classifying overweight and obesity according to the criteria recommended by the National Health Commission in China (24). DN was defined as persistent albuminuria, progressive reduction in glomerular filtration rate (GFR) and hypertension judged by clinicians.

### Laboratory Assay

Dried blood spots were used in the assay of metabolomics, which were prepared from capillary whole blood through 8-h fasting. We measured the metabolites by direct infusion mass spectrometry technology equipped with the AB Sciex 4000 QTrap system (AB Sciex, Framingham, MA, USA). High-purity water and acetonitrile were purchased from Thermo Fisher (Waltham, MA, USA), and utilized as diluting agent and mobile phase. 1-Butanol and acetyl chloride were obtained from Sigma-Aldrich (St Louis, MO, USA). Isotope-labeled internal standard samples of amino acids (NSK-A) were purchased from Cambridge Isotope Labo-ratories (Tewksbury, MA, USA), while standard samples of the leucine were purchased from Chrom Systems (Grafelfing, Germany). In brief, 8.5 mL of venous blood was drawn from each participant at 08:00 to 09.30 h in the morning after 8-h fasting. Laboratory tests were carried out at a special diagnostic laboratory. The level of lipid profiles was analyzed by an automatic biochemistry analyzer (Hitachi 7150, Tokyo, Japan). We also assayed the level of HDL-C and LDL-C by the selective solubilization method (12) (Determiner L-HDL, LDL test kit; Kyowa Medex, Tokyo, Japan).

### Statistical Analysis

Data with the normal distribution was represented by the mean ± standard deviation (SD), or use the median (interquartile range). Categorical variable was in numbers (percentage). Whether there was a difference between DN group and non-DN group were tested separately in male and female. The continuous variable was judged by student’s t-test or separate variance estimation t-test or Wilcox-W test when appropriate; Categorical variable was analyzed by chi-square test.

Binary logistic regression model was established to obtain the odds ratio (OR) and their 95% confidence intervals (CI). Traditional risk factors for type 2 diabetes patients with DN were adjusted using a structured adjustment scheme. We obtained unadjusted OR values ​​and the OR after adjusted age, gender, BMI (<18.5, 18.5–24.0, 24.0–28.0, >28.0 kg/m^2^), duration of diabetes, smoking, drinking, SBP, DBP, TG, LDL-C, HDL-C, HbA1c, UA, SCR, insulin, statins. Restricted cubic splines curve (RCS) is a smoothing curve that can provide more intuitive relationship curve. We chose 4 knots in RCS (25). We have used it to obtain cutoffs for metabolites related to the risk of developing diabetes (26). We selected a cut-off point by visual checking of the curve where the odds of DN changed.

We repeated logistic regression analysis in males and females respectively to obtain OR values. Additive interaction analysis was used to verify the relationship between gender (male or female) and leucine (in 2 groups by RCS cutoff) for DN. We calculated the relative excess risk due to interaction (RERI), attributable proportion due to interaction (AP) and synergy index (S) to estimate additive interactions (27). RERI>0, AP>0 or S>1 indicates a significant additive interaction (27). To avoid the bias caused by non-incident DN. We exclude the patients who with duration of DN >2 years to check the changes of the effect sizes of leucine for risk of DN.

A *p* < 0.05 was considered as statistically significant. All analyses were performed using R version 3.6 and SAS version 9.4 (Institute Inc., Cary, North Carolina, USA).

## Result

### Description of Study Subjects

The mean age and BMI of 1031 participants were 57.2 years (SD:13.8) and 25.3 kg/m^2^ (SD:3.9). Of them, 46.8% were female. There was a total of 188 patients with DN and 92 of them are women. **Table 1** summarized the selected characteristics of DN and controls by sex. In women, cases tended to be older, had higher BMI and longer duration of diabetes, higher SBP, HbA1c, HDL-C, LDL-C, and SCR, and were less likely to use insulin and statins than patients without DN. In men, patients with DN had longer duration of T2D, higher SCR, UA and were less likely to use insulin than controls.

**Table 1:** Clinical and biochemical characteristics of participants according to the occurrence of diabetic nephropathy.

Variables	Women		*p* *^a^*	Men		*p* *^a^*
	Non-DN (391)	DN (92)		Non-DN (452)	DN (96)	
	Mean/number (SD or %)	Mean/number (SD or %)		Mean/number (SD or %)	Mean/number (SD or %)	
Age (years)	59.16 ± 12.81	60.54 ± 9.8	<0.001	54.75 ± 14.81	57.72 ± 14.33	0.074
Weight (kg)	63.64 ± 10.56	67.11 ± 13.21	0.007	75.72 ± 12.79	75.43 ± 11.04	0.840
Height (cm)	160.00 (156.00, 163.00)	160.00 (158.00, 165.00)	0.235	172.00 (170.00, 175.00)	172.00 (170.00, 175.00)	0.616
BMI (kg/m^2^)	24.83 ± 3.83	25.87 ± 4.36	0.024	25.53 ± 3.83	25.54 ± 3.41	0.977
BMI<18.5	70(17.9%)	24(26.1%)		104(23.0%)	23(24.0%)	
BMI≥18.5and<24.0	150(38.4%)	32(34.8%)		211(46.7%)	37(38.5%)	
BMI≥24.0and<28.0	162(41.4%)	34(37.0%)		122(27.0%)	35(36.5%)	
BMI≥28.0	9(2.3%)	2(2.2%)		15(3.3%)	1(1.0%)	
Smokingyes	25 (6.4%)	8 (8.7%)	0.577	243 (53.8%)	55 (57.3%)	0.604
Drinkingyes	13 (3.3%)	2 (2.2%)	0.811	221 (48.9%)	54 (56.2%)	0.231
Duration of diabetes (years)	6.97 ± 7.44	8.93 ± 7.71	0.024	3.00 (0.00, 10.00)	10.00 (2.75, 14.25)	<0.001
SBP (mmHg)	140.00 (122.00, 155.00)	149.50 (129.50, 174.00)	0.001	138.71 ± 22.46	142.1 ± 22.42	0.179
DBP (mmHg)	80.79 ± 13.52	83.23 ± 12.59	0.116	83.79 ± 13.3	82.25 ± 14.82	0.312
HbA1c (%)	7.53 ± 3.22	8.64 ± 2.68	0.002	7.66 ± 3.07	8.24 ± 3.11	0.096
Triglyceride (mmol/L)	1.22 (0.82, 2.08)	1.52 (1.02, 2.31)	0.089	1.22 (0.82, 2.00)	1.31 (0.89, 1.97)	0.561
HDL-C (mmol/L)	0.90 ± 0.45	1.07 ± 0.52	0.001	0.84 (0.45, 1.06)	0.85 (0.50, 1.07)	0.265
LDL-C (mmol/L)	2.42 ± 1.24	2.79 ± 1.31	0.013	2.25 (1.13, 2.95)	2.42 (1.27, 3.04)	0.505
SCR (µmol/L)	53.51 (43.41, 70.95)	57.55 (45.59, 119.00)	0.028	68.72 (58.00, 91.10)	87.78 (64.41, 314.17)	<0.001
UA (µmol/L)	307.00 (238.00, 445.50)	340.60 (264.25, 532.35)	0.073	345.00 (274.75, 499.00)	391.50 (326.00, 778.00)	0.004
Leucine (µmol/L)	124.87 ± 43.17	112.69 ± 31.61	<0.001	143.18 ± 49.85	137.24 ± 46.56	<0.001
<175µmol/L	349(79.9%)	42(93.33%)		352(77.87%)	100(22.12%)	
Diabetic medications						
Acarbose	129 (33.0%)	43 (46.7%)	0.018	150 (33.2%)	42 (43.8%)	0.064
Metformin	131 (33.5%)	41 (44.6%)	0.061	155 (34.3%)	31 (32.3%)	0.797
Insulin	275 (70.3%)	85 (92.4%)	<0.001	321 (71.0%)	90 (93.8%)	<0.001
Statins	131 (33.5%)	45 (48.9%)	0.008	152 (33.6%)	41 (42.7%)	0.116

Data are mean (standard deviation), median (interquartile range) or n (%). BMI, body mass index. SBP, systolic blood pressure. DBP, diastolic blood pressure. HbA1c, glycated hemoglobin. HDL-C, high- density lipoprotein cholesterol. LDL-C, low-density lipoprotein cholesterol. UA, uric acid. SCR, serum creatinine.

a Based on the t-test, Wilcoxon rank-sum test or χ^2^ test as appropriate.

### The Relationship Between Diabetic Nephropathy and Leucine

**Figure 1:**
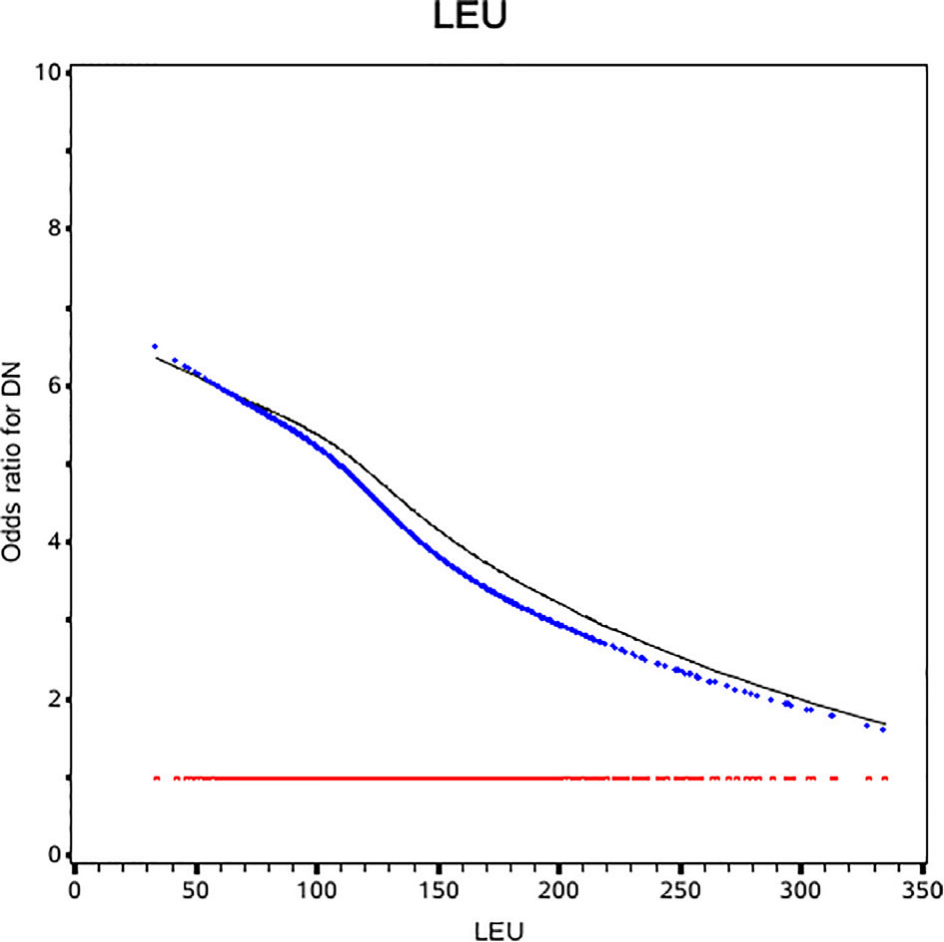
Odds ratio curves of leucine for diabetic nephropathy in Chinese type 2 diabetic patients. The black curve was derived from univariable analysis. The blue one was derived from multivariate analysis that adjusted for age, gender, body mass index, duration of diabetes, smoking, drinking, systolic blood pressure, diastolic blood pressure, low-density lipoprotein cholesterol, high-density lipoprotein cholesterol and triglyceride, glycosylated hemoglobin, urinary creatinine, serum creatinine, insulin, statins (i.e., the odds ratio for diabetic nephropathy was 1).

The slope of RCS curve has a process from small to large and then small, which reaches its maximum at about 175µmol/L. Among all patients, leucine level of 84.8% patient were below 175µmol/L **(** **Figure 1** **).**

In univariable regression, leucine was inversely associated with the risk of DN (OR: 0.8, 95% CI:0.67, 0.95). After further adjustment for traditional risk factors, the negative association was strengthened in multivariable analysis (OR:0.76, 95%CI: 0.62, 0.92) (**Table 2**).

**Table 2:** Odds ratio of leucine for the risk of diabetic nephropathy.

	OR (95%CI)	*p*
Univariable model		
Leucine, per µmol/L	0.8 (0.67,0.95)	0.012
<175 µmol/L	reference	
≥175 µmol/L	0.59 (0.36,0.97)	0.036
Multivariable model1		
Leucine, per µmol/L	0.8 (0.67,0.96)	0.016
<175 µmol/L	reference	
≥175 µmol/L	0.61 (0.37,1.01)	0.045
Multivariable model2		
Leucine, per µmol/L	0.76 (0.63,0.92)	0.006
<175 µmol/L	reference	
≥175 µmol/L	0.56 (0.33,0.94)	0.044
Multivariable model3		
Leucine, per µmol/L	0.76 (0.62,0.92)	0.006
<175 µmol/L	reference	
≥175 µmol/L	0.58 (0.34,0.99)	0.038

Multivariable Model 1 was adjusted for age, gender, body mass index, duration of diabetes, smoking, drinking.

Multivariable Model 2 was adjusted for variables in Model 1 and concentrations of systolic blood pressure, diastolic blood pressure, triglyceride, low-density lipoprotein cholesterol, high-density lipoprotein cholesterol, glycosylated hemoglobin.

Multivariable Model 3 was adjusted for variables in Model 2 and concentrations of uric acid, serum creatinine, use of insulin, use of statins.

### Interaction Between Leucine and Gender

**Figure 2:**
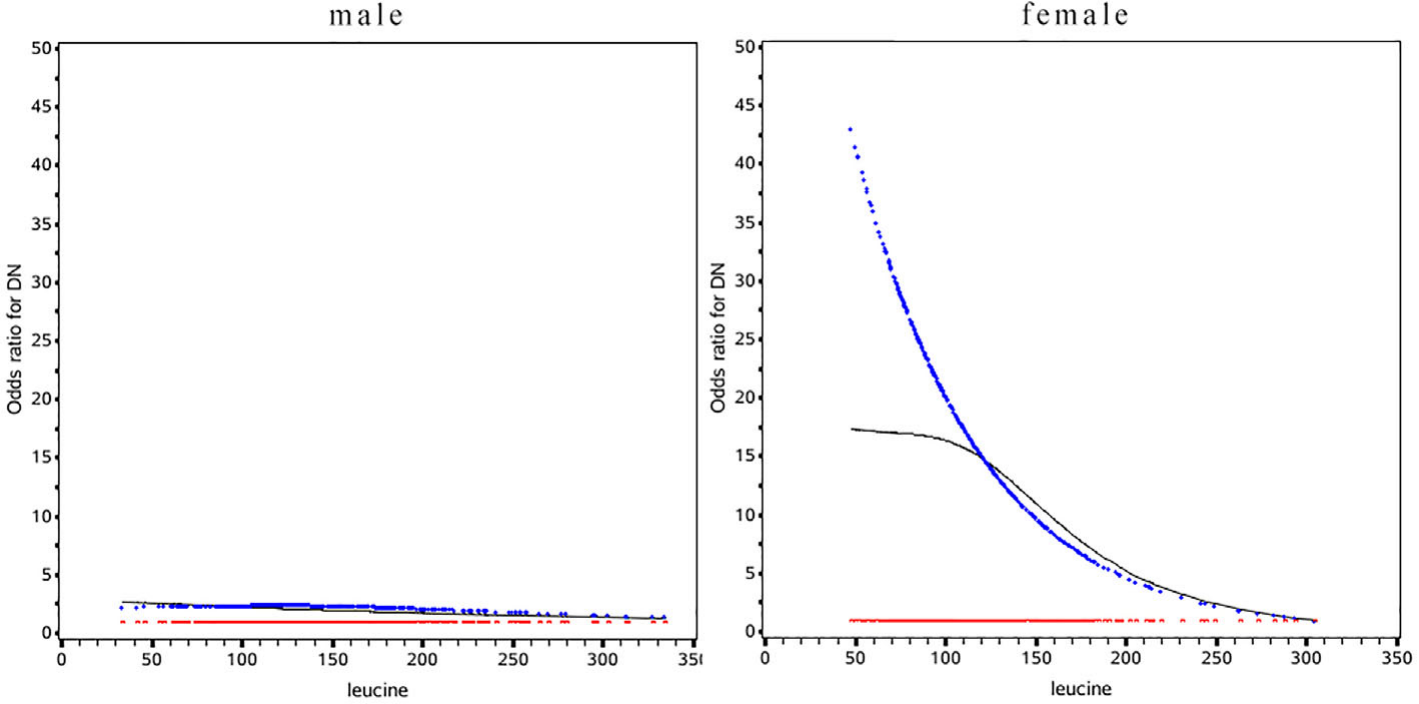
Odds ratio curves of leucine for diabetic nephropathy in Chinese type 2 diabetic patients in different gender. The black curve was derived from univariable analysis, the blue one derived from multivariate analysis that adjusted for age, gender, body mass index, duration of diabetes, smoking, drinking, systolic blood pressure, diastolic blood pressure, low-density lipoprotein cholesterol, high-density lipoprotein cholesterol, triglyceride, glycosylated hemoglobin, urinary creatinine, serum creatinine, insulin, statins(i.e., the odds ratio for diabetic nephropathy was 1).

Leucine was negative associated with the risk of DN in diabetes patients in the female population (OR: 0.51, 95%CI: 0.41, 0.79) while the relationship was not significant in male **(** **Table 3** **)**. In female the risk of DN was decrease rapidly until around 175µmol/L of leucine and then started to relatively flat afterwards. In male, the association between BMI and mortality disappeared **(** **Figure 2** **)**. Using 175 as a cutoff value of leucine, leucine (<175 or ≥175 µmol/L) and gender (male or female) had a significant additive interaction for DN (AP: 0.90, 95%CI: 0.18–1.62; RERI: 0.60, 95%CI: 0.08–1.12; and S: 0.36, 95% CI: 0.11– 1.20).

**Table 3:** Odds ratio of leucine for the risk of diabetic nephropathy in different gender.

	Female (n=483)		Male (n=548)	
	OR(95%CI)	*p*	OR(95%CI)	*p*
Univariable model				
Leucine per µmol/L	0.71(0.55,0.93)	0.008	0.88(0.7,1.11)	0.274
<175 µmol/L	reference		reference	
≥175 µmol/L	0.28(0.08,0.92)	0.037	0.76(0.43,0.34)	0.329
Multivariable model1				
Leucine, per µmol/L	0.7(0.53,0.91)	0.009	0.90(0.71,1.14)	0.380
<175 µmol/L	reference		reference	
≥175 µmol/L	0.29(0.09,0.98)	0.045	0.78(0.44,1.38)	0.394
Multivariable model2				
Leucine, per µmol/L	0.59(0.43,0.81)	0.001	0.90(0.71,1.12)	0.399
<175 µmol/L	reference		reference	
≥175 µmol/L	0.26 (0.07,0.87)	0.030	0.75(0.42,1.36)	0.345
Multivariable model3				
Leucine, per µmol/L	0.57(0.41,0.79)	<0.001	0.92(0.71,1.18)	0.493
<175 µmol/L	reference		reference	
≥175 µmol/L	0.24(0.07,0.83)	0.024	0.81(0.44,1.48)	0.489

Multivariable Model 1 was adjusted for age, body mass index, duration of diabetes, smoking, drinking.

Multivariable Model 2 was adjusted for variables in Model 1 and concentrations of systolic blood pressure, diastolic blood pressure, triglyceride, low-density lipoprotein cholesterol, high-density lipoprotein cholesterol, glycosylated hemoglobin.

Multivariable Model 3 was adjusted for variables in Model 2 and concentrations of uric acid, serum creatinine, use of insulin, use of statins.

### Sensitivity Analysis

After excluding 10 patients who had DN for more than 2 years, the effect size was larger. In the multivariable analysis, the OR value became 0.74 (0.61, 0.91), and the OR value in the female population changed to 0.56 (CI: 0.40, 0.77).

## Discussion

So far, only a limited number of studies have reported the relationship between leucine and DN, and more research focused on the relationship between leucine and diabetes or insulin resistance. For example, the classic Framingham’s 12-year cohort study showed leucine was relevant metabolites in developing diabetes and confirmed the result in the Malmo cohort (28). On the contrary, increased levels of leucine reduced the risk of diabetes in a healthy Japanese population with lower BMI (29). Newgard’s research shows that BCAA contributes to development of obesity-associated insulin resistance, with no significant relationship in lean subjects (15, 30). Only a few population researches investigated the association between leucine and DN. The cross-sectional metabonomic studies was found that patients with DN or albuminuria have lower plasma leucine levels than those only with diabetes (31, 32). And in a case-cohort study, leucine was inversely associated with risk of mortality of vascular disease in individuals with type 2 diabetes (33). In the present study, we found that patients with DN was indeed more likely to have a lower leucine level. And low plasm leucine level was associated with high risk of DN in total subject diabetes patients. Furthermore, we found the relationship between leucine and DN was gender-related. Female with lower plasma leucine had more risk of DN than those who had higher, while in male, leucine did not show significant protective or hazardous effect. In fact, mechanism researches suggested that leucine could activate mTORC1 which control the protein then effect insulin secretion (18, 34–38). Interestingly, mTOR showed a higher expression in females, which explains the gender difference in the effect of leucine on insulin in animal experiments (39–41). Those are consistent with our research results.

Our finding had important clinical and research implications: 1) Discovery of the relationship between leucine and DN will be helpful in diagnosis and disease prediction. Combine a lot of previous researches suggested leucine activates the mTORC1, we speculate that leucine may be a potential predictor of DN; 2) Previously, gender differences in the development of DN were identified. We further clarified the protective effect of leucine on DN only observed in women with diabetes. This observation partly proved that the activation of mTOR by leucine was gender different in the mechanism studies.

The present study has some limitations. Firstly, the cross-sectional study can only prove the statistical association between plasma leucine and DN, but not the exact causal relationship. Due to the lack of research in population, this study provides clues and direction to the further relevant study. Secondly, the levels of leucine are partly influenced by the dietary habits. Since we retrospectively retrieved the data from the hospital’s electronic medical records, the information regarding the diet was not available for further investigation. In order to avoid the bias caused by dietary as much as possible, we used fasting samples and adjusted BMI, LDL-C, HDL-C, and triglyceride; Thirdly, our subjects were hospitalized with T2D, who may have more severe T2D and DN. Thus, caution must be taken when generalizing our findings to other populations; Finally, we did not collect the information of the stage of DN and there may be some changes in metabolism at different stages. We try to exclude patients with DN for more than 2 years in sensitivity analysis roughly estimate of disease stages. The results were still significant in the total study population and female population. Therefore, the report of this study may have a low estimate of the protective effect of leucine on risk of DN.

In conclusion, our study shows that leucine was inversely association with the risk of DN in total subjects and in women with T2D in Chinese. The present study further clarified that this inverse relation between leucine and risk of DN is only observed in women with diabetes, not men with diabetes. It may be a new direction for disease prevention and prediction. Further follow-up studies with stronger causality proving ability are needed to confirm this association in Chinese people and other populations.

## Data Availability Statement

The raw data supporting the conclusions of this article will be made available by the authors, without undue reservation.

## Ethics Statement

The studies involving human participants were reviewed and approved by First Affiliated Hospital of Liaoning Medical University. Written informed consent for participation was not required for this study in accordance with the national legislation and the institutional requirements.

## Author Contributions

Z-ZF and S-LL conceived the project, designed experiments. XG wrote the manuscript and analyzed data. H-HL collected the information and contributed to the writing of this manuscript. XL and X-HQ contributed to the data interpretation. RH collect the information and contributed to the data interpretation and wrote the manuscript and analyzed data. All authors edited the final version of the manuscript. All authors contributed to the article and approved the submitted version.

## Funding

This work was supported by the National Key Research and Development Program of China (2019YFA0802302, 2019YFA0802300), Special Fund of State Key Joint Laboratory of Environment Simulation and Pollution Control. The funders had no role in study design, data collection and analysis, decision to publish, or preparation of the manuscript.

## Conflict of Interest

The authors declare that the research was conducted in the absence of any commercial or financial relationships that could be construed as a potential conflict of interest.
